# Shaping Poultry Meat Quality Attributes in the Context of Consumer Expectations and Preferences—A Case Study of Poland

**DOI:** 10.3390/foods12142694

**Published:** 2023-07-13

**Authors:** Mariola Grzybowska-Brzezińska, Joanna Katarzyna Banach, Małgorzata Grzywińska-Rąpca

**Affiliations:** 1 Department of Market and Consumption, Institute of Economics and Finance, Faculty of Economics, University of Warmia and Mazury in Olsztyn, 10-720 Olsztyn, Poland; margrzyb@uwm.edu.pl (M.G.-B.); malgo@uwm.edu.pl (M.G.-R.); 2 Institute of Management and Quality Sciences, Faculty of Economics, University of Warmia and Mazury in Olsztyn, 10-720 Olsztyn, Poland

**Keywords:** poultry meat, consumer preferences, quality defects, discolouration, hematomas

## Abstract

The optimisation of poultry meat quality attributes at the industrial level requires getting to know the changing customer needs and expectations to justify further measures taken in the future to improve the production process. This article was aimed at the following: (1) to identify and assess consumer expectations and behaviour in relation to the quality criteria for poultry meat offered on the market in Poland, and (2) to identify the significance of the sensory characteristics of poultry meat, mainly variegated colours on its surface, as a criterion for creating the expected quality in a shop. The study results showed that the attributes which significantly lower the quality and commercial value of meat are the defects noticeable on the fillet surface, namely discolouration and hematomas, which are mainly caused during the poultry stunning. The absence of noticeable bruising or hematomas and a uniform colour on the fillet surface are the main attributes of high-quality poultry meat retail cuts, which are expected by consumers. A recommendation for poultry meat producers is that bruising and hematomas need to be eliminated and that the offer should be adjusted to Polish consumers’ expectations about the quality attributes of the meat offered on the market. The elimination of these meat defects will be possible, e.g., through the introduction of innovative technical solutions into the poultry stunning process, which will improve meat quality at its production stage.

## 1. Introduction

Poultry meat is a valuable component of the human diet, and its production over the past several years has been among the fastest developing segments of the meat market worldwide. A steady increase in the production and consumption of poultry meat has been observed in Poland for several years. Since 2014, Poland has been the largest producer and exporter of poultry meat in the European Union [1]. The increase in poultry meat consumption is contributed to by an attractive and affordable price, raw material availability, short preparation time and nutritional and dietary values [2]. Polish consumers declare the following as the quality criteria: nutritional value, sensory characteristics, i.e., colour, tastiness (flavour and aroma), texture (tenderness and hardness), and juiciness [3]. The physical characteristics of poultry meat, i.e., colour or shelf life, are very important in the consumer assessment of quality and determine its consumption [4].

In the process of perceiving a food product through a quality attribute prism, one considers the assumption that the values of a food product are relative, and the opinions on their intensity are formed at two moments [5]: in the shop (when the consumers’ expectations about the possible benefits to be provided by the product are being shaped) and during the actual consumption of the product (the experience of quality). When assessing the quality of food products, consumers definitely make use of impressions based on an assessment of the physical part of the product and assess the benefits resulting from the usefulness of a particular product (according to consumer’s individual needs), which allows them to select a product with an optimal quality [6].

However, it should be noted that the variation in consumer perception and preferences as regards the optimum food product sensory cues is determined by age and gender [7], income, gender, place of residence [8] and gender, age, lifestyle and education [9]. External cues (brand, price, packaging, certificates, manufacturer, etc.) shape different consumer preferences, which is determined by the consumers’ socio-demographic profile that they indicate [10,11].

Meat quality has become a very important attribute for both manufacturers who assess it from an objective perspective (measurable indicators) and consumers who have a more subjective point of view on the perception of quality [12]. Therefore, poultry meat raw material destined for both trade and processing must meet strict consumer quality requirements and be fully identifiable [13,14,15]. The scale and structure of the purchase of poultry meat regarded as a convenient and popular material for consumption will be determined by the functional characteristics of the offered product and the optimum quality attributes. The animal protein preferred in the diets of selected segments of modern consumers is the protein contained in poultry meat. Such a dietary pattern determines the increase in consumer expectations about the offer of this meat in terms of its quality [6,16].

The attributes that significantly lower the quality and nutritional value of poultry meat include the defects visible on the carcass surface/retail cuts, i.e., bruises, subcutaneous haematomas and petechial haemorrhages, which are mainly caused during the process of poultry electric stunning in a water bath [17,18]. These defects are irreversible and difficult to eliminate and account for 20% of defects in a batch of slaughtered poultry. Two commercial quality classes for poultry carcasses and carcass parts are applied on the Polish market, i.e., class A and B [19]. These classes are determined on the basis of the allowable number of defective unit products (with haematomas and discolourations). For consumers searching for poultry meat with particular quality and sensory characteristics [4], the product range was extended to include the meat produced according to the national system of quality assurances for food products (QAFP). As regards the characteristics of high quality “premium” meat, inter alia the following sensory external attributes, as perceived by the consumer, were indicated, i.e., the absence of external hematomas, a colour ranging from light pink to pink, and physical attributes indicating the meat safety and shelf life [20].

The production of poultry fillets with an appearance that is undesirable to consumers results in the product not being purchased, which consequently affects the company’s economics. Manufacturers who have information on the criteria for product choice and on customers’ needs and expectations can take well-founded decisions as part of improving the meat quality management and production control system and creating a competitive market offer [15].

Having considered the source literature research, a study was undertaken with the following aims: (1) to assess how consumer expectations about the quality criteria for poultry meat offered on the market in Poland are met, and (2) to identify the significance of poultry meat sensory characteristics, mainly variegated colours on its surface, as a criterion for shaping quality that is expected and sought for in the shop. The study included the following specific aims:

- To identify the quality characteristics of meat that determine its choice and the quality characteristics that fail to meet consumers’ expectations of quality;
- The determination of consumer attributes of poultry fillet quality assessment;
- To identify consumer responses to the meat characteristics observed during the purchase, which fail to satisfy their quality expectations, with a particular focus on small and large haematomas;
- To identify consumer behaviours as regards poultry fillets with characteristics that fail to satisfy their expectations and to identify high-quality poultry meat attributes.

In order to verify the assumption of the interdependence between the indicated poultry meat characteristics that fail to meet the quality criteria and the socio-demographic characteristics (respondents’ gender and education), the study used the results presented in the source literature [4,7,8,9,10,11,20]. The study examined the following hypotheses:

**H1.**  *Discoloration, contusions and petechial haemorrhages visible on poultry meat are undesirable features and unacceptable by consumers.*

**H2.**  *There is a relationship between the indications of undesirable poultry meat characteristics in the form of blood spots and the socio-demographic characteristics of the respondents.*

**H3.**  *The presence of visible bruises and blood stains on the surface of meat are characteristics of meat that influence consumers’ resignation from buying meat products.*

**H4.**  *Consumer expectations concerning the poultry meat quality attributes include a uniform colour all over the meat surface and the absence of petechial haemorrhages.*

## 2. Materials and Methods

**Figure 1:**
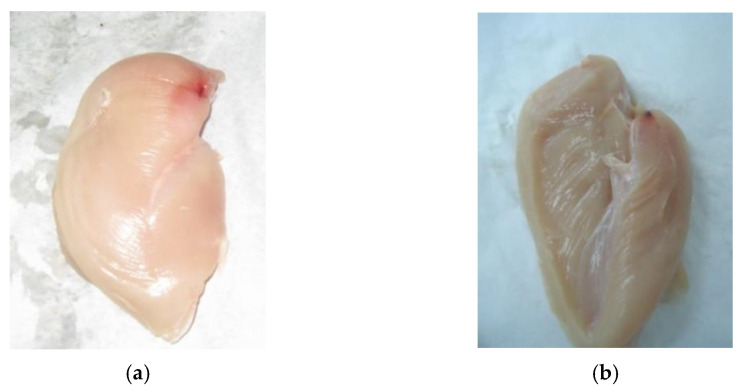
Small haematomas on the surface: (**a**) external, (**b**) internal.

**Figure 2:**
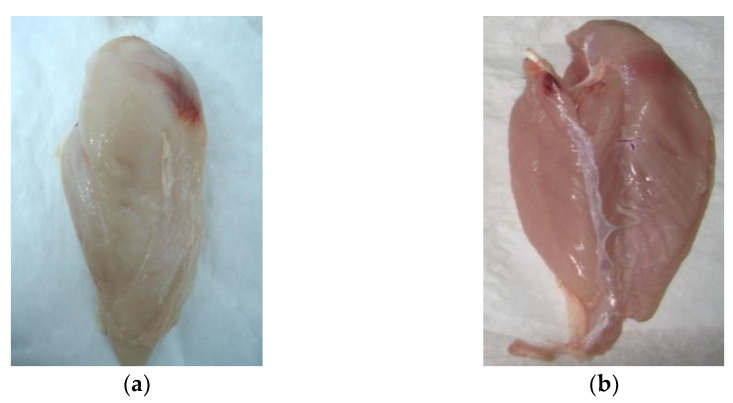
Large haematomas on the surface: (**a**) external, (**b**) internal.

**Figure 3:**
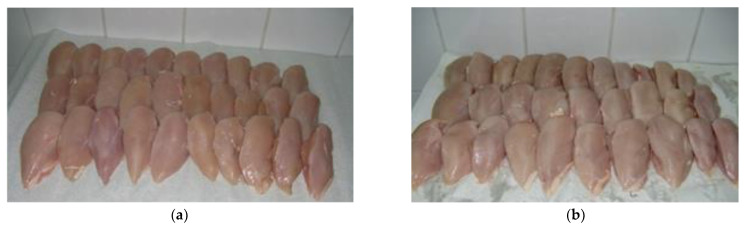
Meat colour: (**a**) dark pink with discolourations, and (**b**) light pink, uniform.

This study was aimed at identifying the behaviours of consumers of the poultry meat (fillets of broiler chickens) offered on the Polish market. The CAPI (Computer-Assisted Personal Interviewing) method was employed in the study. This is an interactive technique to be implemented in direct interpersonal communication with the active participation of an interviewer who reads questions to respondents and then records the responses in an application (an electronic form). The advantage of this method is that it effectively eliminates the interviewing and coding errors resulting, e.g., from the misapplication of filters when conditional questions are provided in the questionnaire. As reported by Boguszewski and Hipsz [21], the results obtained in a study conducted using the CAPI technique are more reliable [22,23]. The CAPI study was preceded by an eye-tracking test aimed at defining the terms used in the questionnaire as regards small and large haematomas. The obtained eye-tracking test results enabled the selection of photographs showing poultry meat imperfections in the form of haematomas small (Figure 1a,b) and large (Figure 2a,b) on the internal and external fillet surface, as well as the of varied colour (dark-pink colour with discolourations—Figure 3a; uniform, light-pink colour—Figure 3b), for the questionnaire.

The basis for the criteria adopted in the study to assess the fillet defectiveness was the considerable scale of the occurrence of quality problems in meat produced in Poland. Similar assessment criteria were also the subject of a study by Banach [24] conducted on turkey meat.

The questionnaire was divided into two main stages. The first stage contained questions related to consumer preferences with regard to the attributes of poultry meat offered on the Polish market. This part examined the respondents’ concerning:

➢ The perception of chicken fillet attributes regarded as meat defects, i.e., dry (D); protruding broken bones (PBB); discolouration on the surface (DS); watery (W); atypical flavour (AF); no defects noticed (NDN); bruising and petechial haemorrhages (BPH); and meat sliminess (MSL),
➢ Responses to the meat defects in the form of small and large hematomas were analysed.

The second stage of the study aimed to identify the respondents’ expectations about high-quality meat, in which the following attributes were considered: uniform colour—Figure 3b (UC); greater health security (GHS); better tastiness (BT); absence of haematomas (AH); and longer shelf life (LSL). Moreover, each part considered the effects of selected socio-demographic characteristics, i.e., the respondents’ gender and education.

In order to select research sample units, the purposive sampling technique was applied. As a non-random technique, it required no random choice procedures and involved the subjective selection of research units. The study was conducted on a group of 793 respondents who were consumers of poultry meat. Since research into the quality of poultry meat and consumer assessments had been conducted in previous studies, the selection of a research group was supported by the researchers’ knowledge and experience [25]. The acquired primary data were substantively verified and 595 questionnaires in which respondents declared the consumption of poultry meat were qualified for the analysis. The population structure by gender was 64% females and 36% males. The largest group comprised respondents aged 18–34 (38%) and 35–44 (35%). Study participants aged above 45 accounted for 27%. Respondents declared mainly secondary (37%) and vocational educational background (35%), while 28% of respondents declared tertiary educational background. The study participants included mainly city dwellers, while 25% were rural area inhabitants.

**Figure 4:**
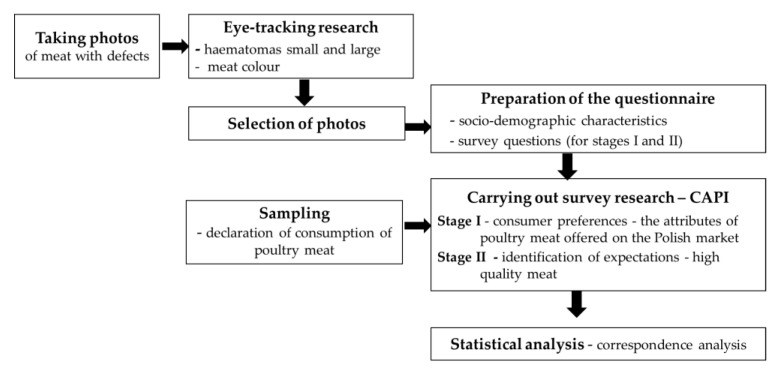
Diagram of the research procedure.

Due to the fact that the research was very complex (multi-stages), Table 1 contains a detailed description of the individual stages of this research process. In addition, a diagram of the test procedure is shown in Figure 4.

Statistical analysis of the obtained data involved the demonstration of the respondents’ preferences related to assessments of meat quality attributes and their purchasing behaviours–the occurrence of small haematomas in poultry fillets. In order to identify these relationships, correspondence analysis was applied, i.e., a descriptive and exploratory technique for analysing contingency tables which enables the presentation of the qualitative variable structure with little loss of information loads [26,27,28,29].

In the literature, you can find many different applications of correspondence analysis in various scientific disciplines [30,31,32,33,34,35]. The analysis made it possible to reconstruct the distance between the socio-demographic characteristics of the respondents and the assessment of the attributes/defects of poultry meat and purchasing behaviours in two-dimensional space. An analysis of the obtained questionnaire survey results was conducted using the Statistica 13.3 program.

The basis for the criteria adopted in the study to assess the fillet defectiveness was the considerable scale of the occurrence of quality problems in meat produced in Poland. Similar assessment criteria were also the subject of a study by Banach [30] conducted on turkey meat.

## 3. Results and Discussion

Understanding the decision-making process of the study respondents who declared the consumption of chicken fillets is an important issue for producers in terms of improving its quality and attractiveness [15], as well as building a competitive advantage for companies [24,36]. Therefore, an attempt was made to identify the most important determinants of the choice of poultry fillets and of the quality defects found in them, as recognised by Polish consumers.

### 3.1. Identify Consumer Preferences as Regards the Attributes of Poultry Meat Offered on the Polish Market

#### 3.1.1. Perception of Chicken Fillets Attributes Regarded as Meat Defects

**Figure 5:**
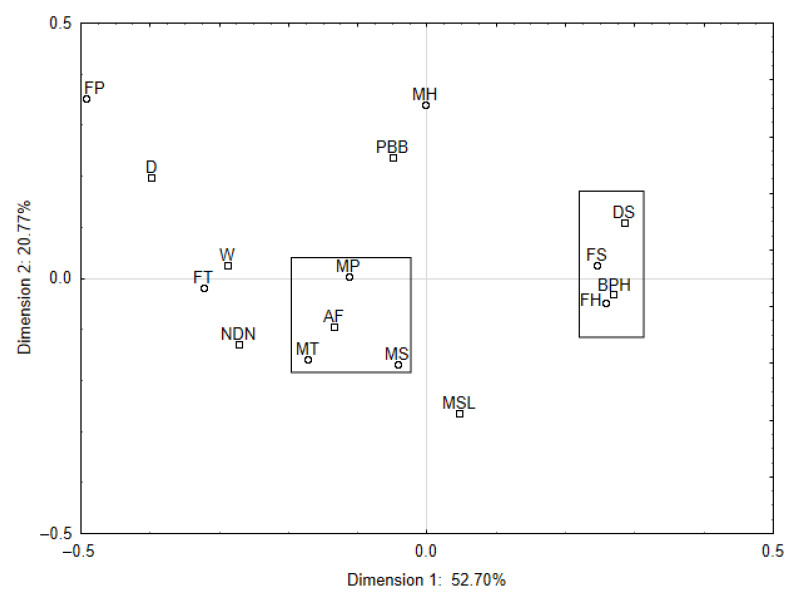
The interdependence between the socio-demographic characteristics (gender and educational background) of respondents and poultry meat defects in a two-dimensional space. Gender/educational background—female: primary (FP), secondary (FS), tertiary (FT), higher (FH); male: primary (MP), secondary (MS), tertiary (MT), higher (MH); meat defects: dry (D); protruding broken bones (PBB); discolouration on the surface (DS); watery (W); atypical flavour (AF); no defects noticed (NDN); bruising and petechial haemorrhages (BPH); meat sliminess (MSL).

A chi-square test of independence ($\chi^{2}\;$ = 4.28, df = 49, *p* = 0.007), determined under the correspondence analysis procedure, indicated the need to reject the null hypothesis of the absence of correlation between the socio-demographic characteristics and the quality defects of poultry meat for the benefit of an alternative hypothesis. The obtained numerical values for the column coordinates indicated that the most important variants of the respondents’ responses for poultry meat defects included wateriness, discolourations on the surfaces and bruising and petechial haemorrhages. It follows from the distribution of points in Figure 5 that the two respondent profiles are predominant: females with a tertiary and secondary educational background who indicate bruising, petechial haemorrhages and discolouration on the surface and males with secondary and vocational educational background, for whom the major meat defect is its inappropriate odour. To sum up, it can be concluded that in the opinion of Polish consumers with secondary educational background, bruising and petechial haemorrhage, as well as abnormal aroma, are recognised as poultry meat defects.

It is, therefore, necessary to search for the causes of the occurrence of defects of raw materials introduced into the market offer and to take preventive measures. The occurrence of meat defects in the form of bruising and petechial haemorrhages is largely determined by the quality of raw materials supplied by the breeders and the poultry-stunning process, i.e., the first stage of meat production [17,18,24]. The inappropriate selection of the electricity parameter values in the process of electric stunning in a water bath (the method most frequently applied in Poland) and the barely humane handling of birds during the unloading and hanging them on a slaughter line are the factors that contribute to the occurrence of quality defects in carcasses and retail cuts [24,37].

Moreover, according to Lines et al. [38] and Sirri et al. [39] the electrical parameters of the stunning equipment, specified in the currently valid Council Regulation (EC) No 1099/2009 [19], contribute to the deterioration in meat quality—an increase in the incidence of large hematomas. In view of the fact that a poultry fillet is recognised as a distinguishing feature of the quality of the meat offered on the market (in terms of the price and nutritional value), and that the haematomas found in them increasingly often reduce their commercial and culinary value, research was undertaken to identify the consumers’ responses to meat with small (Figure 1a,b) and large (Figure 2a,b) haematomas observed during the purchase.

#### 3.1.2. Respondents’ Responses to Meat Defects—Small and Large Hematomas

The appearance of large haematomas in a poultry meat fillet is a very troublesome quality defect that is difficult to eliminate, particularly when they are found inside the muscle. The identification of consumer responses to this raw meat defect will provide important information for meat manufacturers as regards the need to take corrective measures in the stunning process.

**Figure 6:**
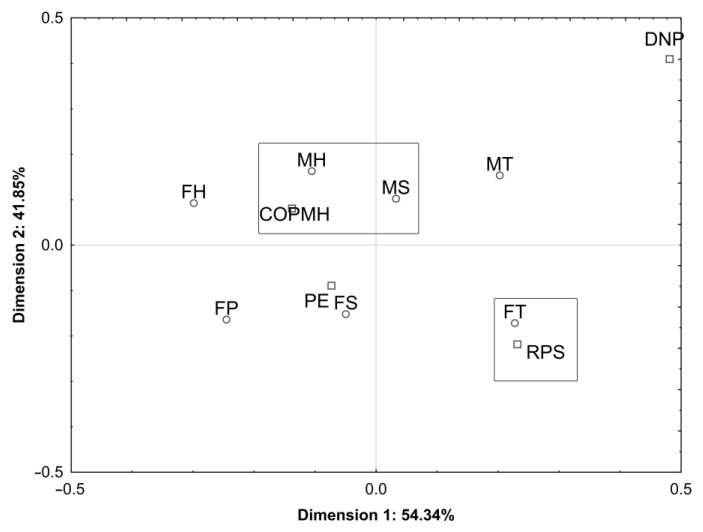
The interdependence between the socio-demographic characteristics (gender and educational background) of respondents and their responses to poultry meat defects in the form of large petechial haemorrhages in a two-dimensional space. Gender/educational background—female: FP—primary, FS—secondary, FT—tertiary, male, MS—secondary, MT—tertiary, H—higher; responses to a defect: PE—request for product exchange; COPMH—cut off the part of meat with haematomas; RPS—return the product to the shop; DNP—defect is not a problem.

The column coordinate values determined under the correspondence analysis procedure indicated that the most frequently declared respondents’ attitude towards the purchase of meat with large haematomas was the return of the product to the shop (r = 0.983) and cutting off the part of the meat with haematomas (r = 0.971). On the other hand, the least frequently declared attitude in the case of this defect was a request for product exchange (r = 0.739). A two-dimensional analysis of the relationships between the respondents’ behaviours in relation to the purchase of poultry meat with large haematomas (Figure 6) enabled the explanation of this relationship in 96.19% (dimension #1: 54.34%; dimension #2: 41.85%). When purchasing meat with defects in the form of large haematomas, it was observed that females with secondary and primary educational background showed the most typical behaviour and requested the fillet exchange, while females with vocational educational background declared the return of the product to the shop. Males with secondary and tertiary educational background indicated that the defect should be removed by cutting off the part of the meat with haematomas.

**Figure 7:**
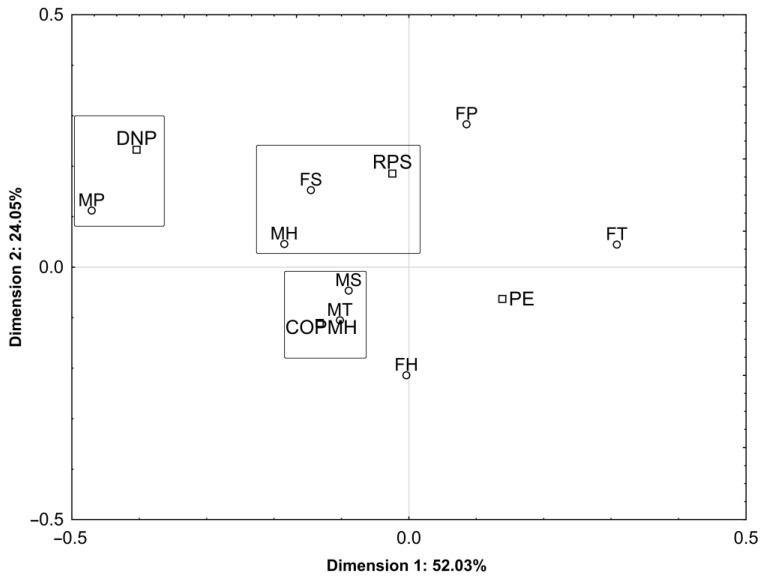
The interdependence between the socio-demographic characteristics (gender and educational background) of respondents and their attitudes (responses) towards a poultry meat defect in the form of small petechial haemorrhages in a two-dimensional space. Att responses to a defect: PE—request for product exchange; COPMH—cut off the part of meat with haematomas; RPS—return the product to the shop; DNP—defect is not a problem.

The presented two dimensions of respondents’ behaviours in relation to poultry meat defects of small haematomas (Figure 7) explain this relationship by 76.08% (dimension #1: 52.03%; dimension #2: 24.05%). The females with vocational and primary educational background and the males with primary educational background are not bothered with the defect in the form of small haematomas, and when it occurs, they most frequently return the product to the shop. Males with vocational and secondary educational background request the exchange of the product or cut off and discard the part of the meat with haematomas.

The obtained study results concerning poultry meat consumer responses to the occurrence of a defect in the form of haematomas (irrespective of their size) in the poultry fillet demonstrated that respondents most frequently declared that they cut off the defective part or requested a product exchange. Therefore, this defect reduces the functionality and use-value of the product. In the assessment of customer’s satisfaction, such a response to the offer does not promote the building of the purchasers’ loyal market behaviours and may even result in the loss of customers. When a defective product (in the customer’s opinion) is placed on the market, the cost is borne by the supplier. Most often, this means the loss of a customer, or a reduction in the price of a product that is regarded as having no full value. It is, therefore, advisable to take care of the quality of the raw materials supplied and to apply the appropriate parameters of the equipment used in the poultry-stunning process. Therefore, the hypothesis that the occurrence of petechial haemorrhages in meat determines Polish consumers’ choices, and is the reason for not buying it, was confirmed.

According to researchers [24,40], in the case of poorer-quality raw materials, the process of electric stunning (device type) contributes to an increased incidence of quality defects in the form of bruises and haematomas in meat. Therefore, the hypothesis that the occurrence of petechial haematomas in meat determines the Polish consumers’ choices and is the reason for abandoning a purchase, was plausible.

Taking measures by manufacturers to improve the meat quality by reducing the scale of incidence of large haematomas may indirectly contribute to extending the meat shelf life, improving the technological quality and reducing the adverse impact on the environment. Banach [24] demonstrated that the use of an innovative, originally developed OIRK device [41,42] helps achieve the average production of defect-free turkey fillets at a level of 75% of the product batch being assessed. A similar level (80%) of haematoma-free meat production was achieved by Lambooij et al. [43], who stunned poultry by the “only-head” method. Changes resulting in the application of an alternative poultry stunning technique, i.e., gas stunning, or continuing to apply the electric stunning technique in individual Member States, will be dependent on the costs of meat production/manufacturing and on consumer demand [17]. If both the profitability and competitiveness of products in the long term are determined by the level of their quality, the need for designing quality products and implementing innovative devices determining the meat product quality is justified.

Therefore, the basic element of the process of designing a new, high-quality product is to identify the quality attributes that are desirable and expected by the consumer and subsequently to translate consumer needs into the technological specificity of the new product’s added value.

### 3.2. Respondents’ Expectations of High-Quality Meat

When describing high-quality food products, the modern consumer most often pays attention to their health security (the absence of chemical and microbiological contamination), the absence of defects, sensory appeal (general appearance, colour, flavour, aroma, tenderness, and juiciness), nutritional value, and product availability [3,13]. Product evaluation carried out by the consumer is subjective and does not always address all the above-mentioned meat quality attributes, as it takes into account only those that appear to be capable of meeting a consumer’s individual needs. Therefore, the poultry industry, in order to meet emerging market challenges, must have a good understanding of the customers’ needs and expectations.

**Figure 8:**
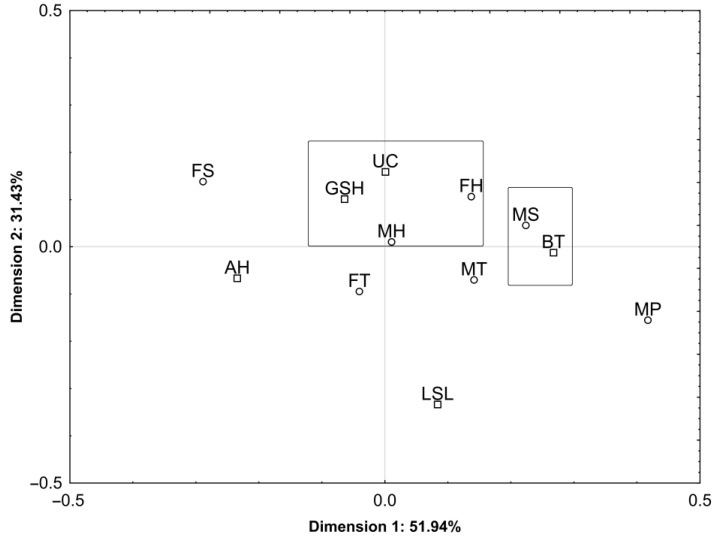
The interdependence between the socio-demographic characteristics (gender and educational background) of respondents and the poultry meat high quality attributes in a two-dimensional space. Attributes: UC—uniform colour; GHS—greater health security; BT—better tastiness; AH—absence of haematomas; and LSL—longer shelf life. Gender/educational background—female: FS—secondary, FT—tertiary, FH—higher; male: MP—primary, MS—secondary, MT—tertiary, MH—higher; Attributes: UC—uniform colour; GSH—greater health security; BT—better tastiness; AH—absence of haematomas; and LSL—longer shelf life.

Regarding consumer preferences and responses to meat defects in the form of small and large haematomas (presented in the first part of the paper), the respondents were asked a question concerning their expectations toward high-quality poultry meat. The presentation of links between the respondents’ characteristics, i.e., gender and educational background and their behaviours regarding the poultry meat high-quality attributes in the form of a two-dimensional factor space (Figure 8) explain this correlation by 83.37% (dimension #1: 51.94%; dimension #2: 31.43%).

The most typical group was the group comprising male and female respondents with tertiary educational background, for whom, in the assessment of high meat quality, the dominant attributes included a uniform light-pink colour and greater health security. Males with a secondary and vocational educational background perceive the high quality of meat in greater tastiness.

Consumer preferences for a uniform light-pink colour of fillets (Figure 3b) were also demonstrated by Wideman, O’Bryan and Crandall [44]. The phenomenon of abnormal colouring or bicolouration in fillets has an adverse effect on their purchase and may even result in the rejection (complaint) of the product by the consumer [45]. A study conducted by Fletcher [39] demonstrated that the colour of the breast muscles of chickens produced in five commercial facilities varied considerably, and in 7% of the unit packets being assessed (trays, four fillet pieces), at least one fillet was clearly distinguishable in colour from the others. The appearance of moderate-to-extensive white striations visible on the fillet surface reduces consumer acceptance of the fillets, with over 50% of the respondents declaring that they would probably, or definitely, not purchase fillets with this defect type [36].

The recognition of the light-pink colour and the absence of haematomas as the most important attributes of high meat quality by Polish consumers confirmed the assumed hypothesis, and indicate the validity of taking quality-promoting measures and providing information on the added values being designed [46]. On the Polish market, such measures were taken in the production of meat with the QAFP mark, with strictly defined high-quality attributes and a guarantee of safety along the entire production chain. Despite the popularisation of the QAFP system and the measures taken to promote the qualitative values of this meat, the degree of awareness of the QAFP system among consumers on the domestic market determined, to a small extent, the purchase of high-quality poultry meat in terms of the absence of haematomas [47]. The petechial haemorrhage demonstrated in the study, which are still perceived during the purchase of meat as troublesome to customers, indicate the topicality of the problem, and the need for an educational campaign aimed at consumers. Continuing and taking new strategy measures to differentiate meat products in terms of the quality attributes preferred by customers, combined with the implementation of animal-welfare-promoting measures (in line with the Green Deal strategy), will allow the promising market potential to be made use of [4].

## 4. Summary and Conclusions

As a food product, poultry meat needs to be characterised by certain qualities which, if specified as desirable, will promote its consumption. The quality of poultry meat at the point of sale is determined by the carcass and meat colour, the degree of muscling, visible fat content, aroma, and freshness; at the moment of consumption, these characteristics include the nutritional value and sensory characteristics (colour, texture, tastiness, and juiciness). Polish consumers believe that the nutritional value and sensory characteristics are important determinants of poultry meat quality and the product appearance is of great importance in the purchasing process. The scale of poultry meat consumption confirms the Polish consumers’ preferences, and the significance of expectations and attributes of poultry meat consumer quality is a very important area of research that is of significance to the shaping of the competitiveness of the offer by producers and processors.

1. The main characteristics of the expected quality of poultry meat are those interpreted by consumers as sensory (appearance–colour) and functional attributes (nutritional value and protein content).
2. Analysis of the consumer assessment results demonstrated that, in the respondents’ opinion on the quality of the meat offered on the poultry meat market, the most frequent characteristics that are undesirable and not accepted by consumers include discolourations, bruises and petechial haemorrhages which are clearly visible on both the external and internal fillet surface. Hypothesis No 1 was positively verified.
3. When buying poultry meat, consumers pay particular attention to the absence of visible bruises and blood spots on the meat surface and to a uniform colour. Respondents who have bought a fillet with a defect of petechial haemorrhages, irrespective of their size, request a product exchange or cut off the part of the meat with haematomas. Hypothesis No 3 was positively verified. The respondents’ attitudes towards meat with defects, including meat with haematomas, are significantly affected by gender and education (socio-demographic characteristics). The obtained relationships confirmed Hypothesis No 2.
4. The expectations declared by consumers with regard to the quality attributes for the poultry meat offered on the market indicate the need for improving its overall appearance by eliminating petechial haemorrhages from the fillet and ensuring a uniform colour all over its surface. Hypothesis No 4 was positively verified.

The obtained research results indicate that consumers prefer high-quality meat, which is manifested, among others, by its colour, smell or freshness. It is worth emphasizing that the quality of meat products is influenced, among others, by the proper maintenance of animals, their feeding and handling at every stage, i.e., production, transport, ante-mortem and post-mortem procedures. The methods of obtaining raw materials are subject to systematic changes, which are the result of the use of new technologies and technical progress. An element of the comprehensive technological transformations taking place in meat production is the welfare of animals subjected to slaughter. The increase in consumer awareness observed in recent years is a determinant of dynamic changes for meat industry producers, which is also a challenge. In order to meet the growing demands, it is not enough that the taste qualities are the only feature that a high-quality product should have. Important information that the consumer is looking for is also the origin of the raw material and the treatment of animals from which this raw material comes.

## Figures and Tables

**Table 1:** Stages of the research process.

Stage of Proceedings	Description
The recruitment of respondents	Recruitment of participants of the customer survey in stores with poultry meat offer. Involve students in recruiting volunteers and gather contact information.
The selection of respondents	The elimination of respondents who were not consumers of poultry meat.
The selection of the test sample	The determination of the population of 739 respondents, taking into account the structure by gender. The characteristics of the recruited population of respondents consuming poultry meat were assessed and a comparison of the population of respondents recruited for the study with the characteristics of Poles consuming poultry was performed.
The construction of research tools	The classification of hematomas. The identification of types of hematomas present in batches of poultry meat which is on offer on the market: production plants—interviews with commodity experts; retail—interviews with sellers.
The standardization of perception of hematoma size	Stage I—the preparation of photos presenting the types of hematomas occurring in the production process. Stage II—the recruitment of experts from the group of consumers declaring the consumption of poultry meat to determine (standardize) the diagnosis of hematomas by consumers participating in the study. Stage III—the preparation of photos for the pilot study.
Designing an experiment	Stage I—as a result of the pilot study, the most commonly used names in relation to the hematomas presented in the pictures were selected. Stage II—the preparation of a questionnaire, which was used in a pilot study on a group of 40 respondents recruited from the market. Stage III—after the correction of the questionnaire, the assignment was carried out in a group of 739 respondents. After substantive verification, 595 correctly completed questionnaires were accepted for analysis.
Research implementation	Carrying out interviews with poultry meat consumers.

Source: own study.

## Data Availability

The data presented in this study are available on request from the corresponding author.
